# Rapid mental health screening in conflict zones: a translation and cross-cultural adaptation into Arabic of the shortened Revised Child Anxiety and Depression Scale (RCADS-25)

**DOI:** 10.1186/s13031-021-00386-1

**Published:** 2021-07-01

**Authors:** Jon D. Perkins, Julieta Alós

**Affiliations:** 1 PMARC, Edinburgh University, Edinburgh, UK; 2 Department of English Literature & Linguistics, Qatar University, P.O. Box 2713, Doha, Qatar

**Keywords:** Arabic, Translation, RCADS, Depression, Anxiety

## Abstract

**Background:**

During conflict, children and adolescents are at increased risk of mental health problems and in particular, anxiety and depression. However, mental health screening in conflict settings is problematic and carries risk making the need for fast, easy-to-administer, screening instruments paramount. The shortened version of the Revised Child Anxiety and Depression Scale (RCADS-25) is one method of rapidly assessing anxiety and depressive symptoms in youths. This self-report questionnaire demonstrates good internal consistency and diagnostic capacity in clinical and non-clinical populations. Nevertheless, few studies have tested the psychometric properties of translated versions of the RCADS-25 limiting its applicability worldwide.

**Objectives:**

To expand the reach and utility of the RCADS-25, the present study sought to develop an Arabic version of the instrument (RCADS25-Arabic) and to explore its reliability and underlying factor structure. In light of changes to DSM classification, the effects of removing indicator variables for obsessive-compulsive disorder on the psychometrics of the RCADS25-Arabic were also explored.

**Method:**

The scale was back translated into Modern Standard Arabic and administered to 250 Arabic speaking schoolchildren between 8 and 15 years of age in Syria. Mean and standard deviation were used to characterise the sample and summarize scores. The reliability and factor structure of the RCADS25-Arabic was explored using confirmatory factor analysis.

**Results:**

Females were 127 and mean age was 12.11 ± SD 2.35. Males scored lower on anxiety (*M* 15.05 *SD* ± 8.0, *t*(248) = − 3.15, *p* = .003, d = 0.39) and internalizing factors (*M* 26.1 *SD* ± 13.1, *t*(248) = − 2.36, *p* = .0160, d = 0.31) with no statistical gender difference recorded for depression (*t*(248) = − 1.27, *p* = .202). Fit statistics were good for two- and one-factor solutions (χ^2^/*df* = 1.65, RMSEA 0.051, CFI .91, TLI .90 and χ^2^/*df* = 1.64 and RMSEA 0.051, CFI .91 and TLI .89 respectively). DIFFTEST showed no significant difference between models (χ^2^_diff_ (1) = 0.03, *p* < 0.86) indicating a one-factor (internalizing) solution was preferable. No improvement in scale integrity was found after deleting obsessive-compulsive disorder items.

**Conclusion:**

The RCADS25-Arabic is useful for rapid screening of depression and anxiety but is better used to identify a one-factor internalizing construct. Obsessive-compulsive disorder items should be retained in the RCADS-25.

## Introduction

In a recent global meta-analysis, the pooled prevalence of mental disorders in children and adolescents was 13.4% (CI 95% 11.3–15.9) [37]. Anxiety and depression were two prominent psychopathologies reported in that study, which are related to other mental health issues such as; post-traumatic stress, substance abuse or aggressive behaviours [9]. During times of conflict, anxiety and depression have been shown to increase in children and adolescents [7]. These authors report pooled estimates of 43, and 27% for each disorder respectively, although follow-up studies show conflict related mental health can improve in a short period of time [38]. The Middle East and North Africa are areas that continue to suffer significant turmoil and these regions have elevated levels of mental health burden [2, 36, 43]. Surveying vulnerable populations in these areas can be complicated by on-going fighting or access restrictions from state or military actors. These problems highlight the necessity for quality diagnostic and screening tools in Arabic that are quick to administer and easily available to distribute. The usefulness of Arabic mental health screening tools is further emphasised by the large numbers of Arabic speaking refugees living in other parts of the world, with many having suffered significant psychological distress in their home countries, during flight or in adapting to new cultures [45]. In a study with over 1,000 Syrians resettled in Sweden, for example, 40.2% showed depressive symptoms and 31.8% symptoms of anxiety above threshold [44].

Adapting mental health screening tools across cultures and contexts is problematic. A systematic review of 26 different child and adolescent screening instruments found that none were convincingly satisfactory with regards to cross-cultural validity [41]. Multiple factors contribute to this finding such as variation in understanding, expression and responses to mental health between cultures [30]. In addition, translation is rarely a case of substitution as words often have alternate meanings or different connotations in the target language [3]. Despite these difficulties a number of child mental health screens have been adapted to Arabic (e.g. the Children’s Depression Inventory 2) [32] or the Screen for Child Anxiety Related Emotional Disorders (SCARED) [23]. However, their usefulness can be limited by factors such as; measuring only a single mental health dimension, not being based on *Diagnostic and statistical manual of mental disorders* DSM-V [6] classifications, measuring only a limited age group, being too costly or too long to complete where sometimes brief assessment is required (e.g. in conflict zones).

### The Revised Child Anxiety and Depression Scale

A freely available self-report questionnaire used worldwide is the Revised Child Anxiety and Depression Scale (RCADS) [11, 12]. The full version has 47 questions pertaining to symptoms of depression and the five dimensions of anxiety specified by the DSM-IV [5]: generalized anxiety disorder, separation anxiety disorder, social phobia, panic disorder and obsessive-compulsive disorder. However, the more recent DSM-V [6] classifies obsessive-compulsive disorder as a distinct category (Obsessive-Compulsive and Related Disorders). In addition to overall anxiety and depression scores, the RCADS captures a unidimensional factor (anxiety and depression together) which is a higher-order construct described as an ‘internalizing disorder’ or ‘general negative affect’ [10, 14]. The scale is aimed at youths in grades 3–12 (ages 8–18 years) and asks participants to rate statements such as *“I would feel afraid of being on my own at home”*, using a 4-point Likert scale ranging from 0 (never) to 3 (always). Clinical significance is determined by t-scores after raw totals are adjusted in relation to gender and school year.

As 47-items are cumbersome for brief assessment, a shortened 25-item version (RCADS-25) including 15 anxiety and 10 depression questions was produced [14]. As with the larger scale, child (RCADS-25) and parent versions (RCADS-25-P) were developed which have been validated in clinical and non-clinical populations [13, 35]. The brevity of the RCADS-25 makes it ideal for screening children in a fast and efficient manner, and therefore, for use in conflict zones. However, the RCADS-25 has yet to be validated in Arabic, limiting its reach in areas of the world where it is greatly needed.

A further aspect to this work relates to the reclassification of obsessive-compulsive disorder (OCD) in the DSM-V. This reclassification has generated considerable debate among investigators primarily around whether or not there is sufficient symptomology, treatment response, comorbidity, neural and genetic foundations, to classify OCD as a distinct entity [1, 25]. Regardless of this debate, the reclassification has implications for anxiety screening and diagnostic tools many of which carry OCD related items (including the RCADS).

The aims of this study were to create an Arabic version of the children’s RCADS-25 and to test its reliability and factor structure in a cohort of Arabic speaking children living in Syria. In addition, this work explored how changes in the DSM-V classification of anxiety (i.e. removing OCD items) affected the psychometric properties of the scale.

## Methods

### Translation

The RCADS-25 was translated using aspects of the protocol suggested by Sousa & Rojjanasrirat [39]. Professional translators, with the assistance of psychologists familiar with mental health assessment carried out the interpretation. The English version was forward translated into Modern Standard Arabic (MSA) before being back-translated by a different team and compared. Several translation issues arose; in written Arabic, the words for anxiety and worry are the same, “قلق” (*qalaq*), whereas in spoken Levantine Arabic, قلق means “insomnia”. Interpretation, therefore, is dependent on relevance and context [3]. In addition, when the word is used to specify anxiety, it refers to an ‘adult’ concept that is unlikely to be understood by children. Hence, in some cases, items referring to ‘worry’ were translated to the more closely related “خشية” (*ḵhašya*) “*fear of the likelihood of something happening*”. Three statements were amended in this way; item 2, “*I worry when I think I have done poorly …*” , item 5, “*I worry that something awful will happen to someone in my family*” and item 20, “*I worry that I will suddenly get a scared feeling when there is nothing to be afraid of”*. A further translation issue was that in MSA there is no verb ‘to be’ thus the statement *“I am tired a lot”* (item 21) was modified to *“I* *feel tiredness* *a lot”.* Discrepancies between versions were resolved through collaboration between teams and the Arabic scale amended before administration.

### Participants and protocol

Children from grades 3–10 (ages 8 to 15) completed the child version of the RCADS25-Arabic. We aimed to enrol a minimum of 20 children from each grade, (10 males and 10 females). Participants were recruited from schools in Damascus Governate, Syria. Ethical approval was secured from the Faculty of Education, Damascus University. Headteachers in 10 randomly selected schools that were stage 1 and 2 in the Syrian curriculum, were approached to request participation. Three schools agreed and letters were sent home to gain written informed consent from parents/guardians and children who wished to take part. No incentives were given for participation. The RCADS25-Arabic was administered on paper in the classroom with a teacher and psychologist present and demographic information was collected simultaneously. The survey was explained and children were encouraged to answer all questions as best they could. Children were informed of their right not to answer questions they were uncomfortable with and to withdraw from the study at any time. Information was given regarding local and web-based mental health providers in case taking part in the study raised concerns. Completion of the questionnaire took approximately 10 min.

### Data analyses

Thirty-one out of 250 participants (12.4%) had one or more missing responses. Missing data were addressed by prorating remaining items in the scale using multiple imputation method in SPSS version 22 [26]. For the sample size used in this work, five imputations were employed [48]. Independent-samples t-tests (two-tailed) were run in SPSS to investigate gender differences in mean raw scores for each scale with significance set at *p* < 0.05. To examine the extent to which different scales represented individual phenomena, Cronbach’s alpha coefficients were calculated with scores ≥7.0 used to indicate acceptable reliability [20]. The contribution of each item to respective scales was evaluated with corrected item-total correlations. Performance was based on correlations being ≥.30, <.8, as well as the degree to which item removal affected coefficients (drops of > 10% were considered undesirable) [34].

Factor structure was explored using confirmatory factor analysis (CFA) in Mplus version 6.1 [33]. As a two-factor solution with depression and anxiety as latent factors has been established in studies of the full RCADS (e.g. [13]), we looked to confirm this model. Structure and reliability were also explored with obsessive-compulsive disorder (OCD) items removed. Data was multivariate non-normal so weighted least-squares of mean and variance (WLSMV) was used as an estimator. Latent factor variance was freely estimated after loadings of the first item on each factor was set to one. To explore data fit, four indices commonly reported elsewhere were applied; relative chi-square (χ^2^/*df*), root mean square error of approximation (RMSEA), the Comparative Fit Index (CFI) and the Tucker-Lewis Index (TLI). Cut-off criteria were < 2.0 for relative chi-square, close to 0.5 for RMSEA (with scores < 0.8 satisfactory) and ≥ .90 for CFI and TLI (with ≥.95 indicating excellent fit) [28]. To explain the interaction between indictors and latent factors, standardized factor loadings with a cut-off of value ≥.35 were used [22].

Nested models were compared using DIFFTEST, which produces chi-square statistics of change in model fit. A non-significant finding indicates no difference between models and suggests that the simpler (less constrained) model should be retained.

## Results

### Demographics

Two hundred fifty children took part in the study, 123 males and 127 females (mean age 12.11 ± SD 2.35, range 8–15 years). Statistically significant gender differences were observed in individual scale mean scores as determined by independent-samples t-tests. Males scored significantly lower on anxiety (*M* 15.05 *SD* ± 8.0, *t*(248) = − 3.15, *p* = .003, d = 0.39) and internalizing factors (*M* 26.1 *SD* ± 13.1, *t*(248) = − 2.36, *p* = .0160, d = 0.31), than females (*M* 18.22 *SD* ± 8.2, *M* 30.2 *SD* ± 13.2 respectively) (see Table 1). No statistically significant gender difference was recorded for depression (*t*(248) = − 1.27, *p* = .202). Effect sizes were moderate for both anxiety (*d* = .39) and internalizing factors (*d* = .31).

**Table 1:** T-tests and demographics for children and parents

		Total sample ***n*** = 250			
	Grade	Male (n)	%	Female (n)	%
**School**	3	20	8.0	19	7.6
	4	10	4.0	12	4.8
	5	15	6.0	16	6.4
	6	20	8.0	19	7.6
	7	14	5.6	10	4.0
	8	11	4.4	14	5.6
	9	21	8.4	18	7.2
	10	12	4.8	19	7.6
	Total	123	49.2	127	50.8
**RCADS-25**	**Scale**	**Mean (SD)**	**t (*df*)**	***p*** **value**	**Cohen’s** ***d***
M (123)	Anxiety	15.05 (8.0)	3.15(248)	.003^*^	.39
F (127)		18.22 (8.2)		–	–
M (123)	Depression	10.6 (6.2)	1.28(248)	.202	–
F (127)		11.7 (5.9)	–	–	–
M (123)	Internalizing	26.1 (13.1)	2.36(248)	.019^*^	.31
F (127)		30.2 (13.6)	–	–	–

*M* males, *F* females,*Significant at *p* < .05 level

% = Percentage of total sample (250). Totals under 100% indicate missing data

### Reliability

Cronbach’s alpha was acceptable for all scale dimensions. The lowest score was .71 for depression with anxiety .76 and the internalizing factor .85 (Table 1).

The ranges of corrected item-total correlations in the two-factor model were .24–.49 for anxiety and .28–.42 for depression (see Table 2). Three items fell below .3, items 5 and 12 on the anxiety factor and item 8 on the depression scale. Nevertheless, removal of any item did not improve Cronbach’s alpha. Moreover, for depression, the removal of any item caused Cronbach’s alpha to drop below .70 highlighting the importance of each question to scale integrity. For the one factor solution, corrected item-total correlations ranged from .26–.51. One item was below the .3 cut-off and this was also item 8 on the depression scale.

**Table 2:** Reliability and item level statistics for RCADS25-Arabic

Reliability	Scale	Mean	Items	α	α^**b**^
*N* = 250	Anxiety	16.8 (8.3)	15	.76	.72
*N* = 250	Depression	11.3 (6.1)	10	.71	–
*N* = 250	Internalizing	28.2 (13.5)	25	.85	.83
**Scale**	**Item number in original RCADS-25**	**Factor loading (SE)**^**c**^	**Corrected item-total correlation**^**c**^	**Cronbach’s alpha if item deleted**^**c**^	
Anxiety	2	.39 (.06)^§^	.30	.75	
	3	.40 (.06) ^§^	.32	.75	
	5	.44 (.06) ^§^	.28	.76	
	6	.46 (.06) ^§^	.30	.75	
	7	.48 (.05) ^§^	.32	.75	
	9	.56 (.05) ^§^	.34	.75	
	11	.58 (.05) ^§^	.33	.75	
	12^a^	.62 (.04) ^§^	.24	.76	
	14	.57 (.05) ^§^	.40	.75	
	17†	.49 (.06) ^§^	.43	.74	
	18	.38 (.06) ^§^	.41	.74	
	20	.46 (.05) ^§^	.49	.74	
	22	.59 (.05) ^§^	.48	.74	
	23^a^	.62 (.04) ^§^	.44	.74	
	25	.63 (.04) ^§^	.39	.75	
Depression	1	.38 (.06) ^§^	.37	.69	
	4	.40 (.06) ^§^	.37	.69	
	8	.34 (.06) ^§^	.28	.70	
	10	.64 (.04) ^§^	.34	.69	
	13	.62 (.04) ^§^	.31	.70	
	15	.48 (.05) ^§^	.42	.68	
	16	.47 (.06) ^§^	.39	.68	
	19	.44 (.06) ^§^	.40	.68	
	21	.56 (.05) ^§^	.39	.68	
	24	.53 (.05) ^§^	.42	.68	

α = Cronbach’s alpha. ^a^OCD items, ^b^OCD items removed

§Significant at *p* < 0.001. ^c^Factor loadings, item-total correlations and Cronbach’s alpha if item deleted, derived from two-factor model/sub-scales and includes OCD items

Removing OCD items worsened model parameters. Alpha coefficients for anxiety and internalizing factors reduced, (.72 and .83, Table 1) and a greater proportion of corrected item-total correlations fell below .3 (ranges .21–.48, .27–.47 and .24–.48 for depression, anxiety and internalizing respectively [data not shown]).

### Factor structure

CFA statistics (Table 3) with OCD questions included supported a two-factor solution but fell short of excellent fit; relative chi-square χ^2^/*df* = 1.65 and RMSEA 0.051 (CI, 0.042, 0.059), CFI .91 and TLI .90. Factor loadings ranged from .38–.63 for anxiety and .34–.64 for depression with item 8 on the depression scale the only loading below acceptable cut-off (.34) (Table 2). Fit statistics also supported a one-factor model; χ^2^/*df* = 1.64 and RMSEA 0.051 (CI, 0.042, 0.059), CFI .91 and TLI .89 with factor loadings ranging from .35–.67 (Table 2). The DIFFTEST function in Mplus showed no significant difference in chi-square values between one- and two-factor models (χ^2^_diff_ (1) = 0.03, *p* < 0.86). After omitting OCD questions, fit statistics were decreased for all outcomes (χ^2^/*df* = 1.74, RMSEA = 0.055 CI, 0.045, 0.064), CFI = .89, TLI = .88). Scores remained tolerable but were marginally below conventional cut-offs for some indices.

**Table 3:** *Fit statistics for confirmatory factor analysis models*

RCADS25-Arabic	Fit measures						
	χ^**2**^	***df***	p	χ^**2**^/***df***	RMSEA	CFI	TLI
2 Factor model	450.90	274	0.001	1.65	0.051	.91	.90
2 Factor model (no OCD items)	364.58	209	0.001	1.74	0.055	.89	.88
1 Factor model	451.42	275	0.001	1.64	0.051	.91	.89
**Correlation**	Depression						
Anxiety	0.77						

*N* = 250. *RCADS25-Arabic* Revised Child Anxiety and Depression Scale (Arabic), *χ*^*2*^*/df* Chi-square/degrees of freedom, *RMSEA* root mean square error of approximation, *CFI* comparative fit index, *TLI* Tucker Lewis Index

## Discussion

In the present study, we translated the RCADS-25 into Arabic and investigated its reliability and structure in a sample of Arabic speaking school-aged children. We found good cross-cultural validity in agreement with other works [40]. We attempted to replicate a two-factor solution as described by Ebesutani et al. [13] but a single factor was a better solution for our data. We also explored how the removal of OCD items would influence shortened RCADS scale structure with our data indicating that inclusion was optimal.

In our study, that females scored higher than males for anxiety symptoms is in keeping with the literature [4]. Depression is also commonly elevated in females [47], however, in contrast to other RCADS investigations (e.g. [31]), no significant difference was found in our work. This may relate to the onset of depressive symptoms generally being between 13 and 15 years of age [21], whereas, the majority of our sample was younger than this category. Anxiety is also often known to develop before depression [17] increasing the likelihood of detecting a difference for this disorder and not depression in our sample. The gender findings reported here are consistent with how the RCADS25-Arabic would be expected to function in a cohort of 8–15-year-old children.

Cronbach’s alphas reached acceptable levels for overall and subscale measures although coefficients were lower than reported for the RCADS-47 [31] and parent versions in English of RCADS-25 [13, 35]. However, age is known to influence scale consistency, which may explain the discrepancy between those findings and this study [46]. Coefficients for a latent internalizing factor have typically been found to be high (e.g. [10]) as was the case for our data. It is likely these values reflect an increased number of scale items [42].

CFI nor TLI reached good but not excellent cut-off criteria implying the model could be improved. Nonetheless, for the two-factor solution we report higher scores than previous RCADS-25 research such as Park et al. [35] that describes less robust fit statistics (RMSEA = .088 and CFI = .80 and TLI = .79). Only the depression item 8, which asks children if they have trouble sleeping at night, was below acceptable limits. There is a possibility that this finding reflects actual disturbance caused by the sounds of bombings and rocket fire at night, which are not uncommon to Damascus, rather than psychological distress. The majority of loadings (14/25) fell below .5 for both 1- and 2-factor solutions suggesting many items were low-to-moderate contributors to respective domains and may explain why CFI and TFI scores did not reach higher cut-off thresholds. That the 2-factor structure was not more clearly defined suggests shared variance between anxiety and depression items, which is unsurprising given the overlap of symptoms. In the RCADS for example, the statements “*I feel scared if I have to sleep on my own*” for anxiety and “*I have trouble sleeping*” for depression are naturally associated. In our study the correlation between anxiety and depression was robust (.77) mirroring previous RCADS findings (e.g. [31]) and the broader literature [16, 19]. In the work by Essau and colleagues, they showed a four-fold increase in the likelihood of depression in children and adolescents with anxiety disorder. Anxiety has also been shown to predict depression in survivors of war, a factor that may influence our sample [8]. Explanations for comorbidity include shared genetic origins, common neuroanatomical features, abnormal emotional processing and poor discriminant validity of screening instruments [15, 18, 24]. However, in the latest DSM-V, both disorders are framed within the context of “negative affectivity” and there is ongoing debate about whether depression and anxiety are unique constructs or part of a continuum [29]. A diagnostic category is also in use “anxious depression” but this remains controversial [27]. Nevertheless, our DIFFTEST results are supportive of the idea of a single anxiety-depression concept and research on the RCADS parent version also identified a unidimensional or “internalizing/general negative affect” factor [10, 35].

However, it is important to consider the translation process as a possible contributor to our findings. Items 2 and 5, which were items where ‘worry’ was translated as “خشية” (*ḵhašya*) “*fear of the likelihood of something happening*”, were poorly correlated (.28 and .30) and in the bottom quartile of results for the two-factor solution. Item 2 remained so in the single factor model (.31) with item 5 marginally improving (.36). It is possible therefore, that the translation/cross cultural adaptation process generated some ambiguity in understanding these questions and may have contributed to the single factor solution presenting with stronger statistical fit. The translation of *“I am tired a lot”* (item 21) to *“I* *feel tiredness* *a lot”,* appeared to not be an issue as it contributed well to both models.

We also explored the RCADS25-Arabic with OCD items removed. Two OCD items (12 and 23) were among the highest factor loadings for anxiety (both .62). Item 12 also had the lowest corrected-item correlation hinting that OCD may be an independent subscale as described by DSM-V [6]. Park et al. [35] explored the RCADS without OCD items and reported contrasting outcomes with discriminant reliability improving but factorial statistics diminished. In agreement with the latter finding, our results show inferior alpha coefficients and CFA statistics without OCD items. Our conclusion, therefore, is that OCD items should be retained.

### Study limitations

There were a number of limitations that should be considered when interpreting the results of this study. Only a single data collection was performed and no other mental health assessment tool was used, meaning convergent and discriminant reliability, as well as test-retest reliability, could not be evaluated. Further testing would have strengthened the findings reported here but was beyond the scope and finances of this project. Data was collected from under 16 s limiting the generalizability of our findings to older teenagers. A final consideration is that, as Syria is currently undergoing significant turmoil, it is difficult to determine if our sample is representative of a general or clinical population.

## Conclusions

This study confirms the psychometric properties of the RCADS25-Arabic and highlights its usefulness in screening for internalizing disorder, anxiety and depression in Arabic speaking children between 8 and 15 years of age. Our findings highlight the cross-cultural applicability of internalizing, depression and anxiety constructs and the use of self-report for screening these psychopathologies. Lastly, our findings support the inclusion of OCD items in the RCADS25-Arabic.

## Data Availability

At request of lead author.
